# Does treatment of subsyndromal depression improve depression-related and diabetes-related outcomes? A randomised controlled comparison of psychoeducation, physical exercise and enhanced treatment as usual

**DOI:** 10.1186/s13063-015-0833-8

**Published:** 2015-07-15

**Authors:** Mirjana Pibernik-Okanović, Norbert Hermanns, Dea Ajduković, Jadranka Kos, Manja Prašek, Mario Šekerija, Marijana Vučić Lovrenčić

**Affiliations:** Merkur University Hospital, Vuk Vrhovac University Clinic for Diabetes, Zajčeva 19, 10000 Zagreb, Croatia; Forschungsinstitut Diabetes-Akademie Bad Mergentheim (FIDAM GmbH), Theodor Klotzbücher Strasse 12, 97980 Bad Mergentheim, Germany; Croatian Institute of Public Health, Rockfellerova 7, 10000 Zagreb, Croatia

**Keywords:** Type 2 diabetes, Subsyndromal depression, Diabetes distress, Psychoeducation, Physical exercise, Enhanced treatment as usual, Quality of life, Diabetes self-care, Metabolic control

## Abstract

**Background:**

Elevated depressive symptoms that do not reach criteria for a clinical diagnosis of depression are highly prevalent in persons with diabetes. This study was aimed at determining the efficacy of psychoeducation and physical exercise compared with enhanced treatment as usual on 1-year changes in depressive symptoms, diabetes distress and self-management, and quality of life and metabolic control in type 2 diabetes patients with subsyndromal depression.

**Methods:**

Adult type 2 diabetes patients who screened positively for depression and expressed a need for professional help with mood-related issues were eligible. Exclusion criteria were clinical depression, current psychiatric treatment and advanced diabetes complications. Out of 365 eligible patients 209 consented to either 6 weekly sessions of psychoeducation (A) and physical exercise (B), or to enhanced treatment as usual (C). Computer-generated sequences for block randomisation stratified by gender were used. Depressive symptoms (primary outcome) and diabetes distress, diabetes self-care, metabolic control and health-related quality of life (secondary outcomes) were analysed at 6-month and 12-month follow-up using repeated-measures ANOVAs.

**Results:**

Out of the 74 patients randomised into group A, 66 into B and 69 into group C, 203 completed the interventions, and 179 patients with all 3 assessments were analysed. Depressive symptoms in participants from the psychoeducational, physical exercise and the enhanced treatment as usual groups improved equally from baseline to 12-month follow-up (time versus time x group effect; F = 12.51, *p* < 0.001, η^2^ = 0.07 and F = 0.609, *p* = 0.656, η^2^ = 0.007 respectively), as did diabetes distress and quality of life (all *p* < 0.001), diabetes self-care (*p* < 0.001 to < 0.05), triglycerides, and total cholesterol and LDL-cholesterol (*p* < 0.001).

**Conclusions:**

The employed interventions had comparable positive effects on 12-month psychological and diabetes-related outcomes suggesting that even minimal intervention addressing patients’ diabetes-related problems and concerns had favourable clinical implications and might be sufficient to treat subsyndromal depression. Further investigation is warranted to clarify possible mechanisms of improvement.

**Trial registration:**

Current Controlled Trials ISRCTN05673017

The message on assigning the above mentioned ISRCTN was received on 11 August 2010

**Electronic supplementary material:**

The online version of this article (doi:10.1186/s13063-015-0833-8) contains supplementary material, which is available to authorized users.

## Background

Subsyndromal depression, defined as the presence of depressive symptoms that do not meet full diagnostic criteria for major depression or dysthymia, prevails in diabetic patients with depressive mood disorders. Among the approximately 30 % of diabetic patients who report elevated depressive symptoms, around two thirds do not reach criteria for a clinical diagnosis of depression, implying that 20 % of these patients are affected by subsyndromal depression [1]. However, even mild depressive symptoms involve impaired quality of life [2], and difficulties in self-managing diabetes [3] and achieving desirable metabolic control [4, 5]. Like more severe forms of depression, subclinical depressive symptoms increase the risk for diabetic complications and mortality [6–10].

Findings from the general population indicate low spontaneous remission rates of subsyndromal depression [11]. Recent studies have uncovered predictors of conversion from minor depression into its more severe forms, chronic illness being one of them [12]. The study examining the course of subsyndromal depression in patients with diabetes has revealed a 42 % 2-year incidence of major depression in individuals with baseline sub-threshold symptoms [13]. Subsyndromal depression is likely to be recurrent, causing poor functioning and impaired quality of life in type 2 diabetes patients [14].

Patients who report mild to moderate depressive symptoms frequently also report emotional problems related to diabetes [15–18], suggesting that sub-threshold depression can be envisaged as a generic distress measure that covariates with diabetes-specific distress [19]. This is considered relevant in developing interventions to reduce psychological symptoms and improve diabetes self-management [20]. Treatments addressing both depression-related and diabetes-related problems are supposed to be more effective than treating depression alone [21].

Research on subsyndromal depression treatment in patients with diabetes is scarce, allowing no reliable conclusion on its effects on depression-related and diabetes-related outcomes. Since trials relying on pharmacological treatment have not justified the use of antidepressants in patients with minor depression [22], psychological and behavioural approaches seem to be reasonable treatment options. However, only few studies have examined non-pharmacological treatments aimed at alleviating depressive symptoms. Employing cognitive-behavioural techniques either alone or in a combination with aerobic activity or diabetes self-management education, has yielded mixed results. An uncontrolled study has reported short-term improvement in both depressive symptoms and glycaemia [23], whereas two recent randomised controlled trials have not demonstrated any improvement in glycaemic control [24, 25]. Effects of non-pharmacological interventions on other diabetes-related indicators including lipid profiles, body weight, adherence to diabetes regimen, diabetes complications, and quality of life have not been investigated sufficiently [26]. Critical assessment of behavioural interventions to reduce depressive symptoms and empower diabetes self-management has been appraised to require further research based on sufficiently large samples, long-term follow-up of effectiveness, and development of interventions that might provide benefits to both physical and mental health [27].

This study explored the significance of treating subsyndromal depression in type 2 diabetes patients while examining the effects of three behavioural interventions – psychoeducation, physical exercise and enhanced treatment as usual – on depressive symptoms, diabetes distress, diabetes self-management, health-related quality of life and metabolic control at 1 year. Our hypothesis was that a psychoeducational course and physical activity intervention, both consisting of 6 weekly 90-minute group sessions would be superior to enhanced treatment as usual, consisting of one 90-minute re-educational session, in improving psychological and diabetes-related outcomes.

## Methods

The study was carried out at the Vuk Vrhovac University Clinic for Diabetes in Zagreb. Data on potential study participants were extracted from the patient registry [28] according to criteria of having had type 2 diabetes for at least 1 year, being aged between 18 and 65 years, and having had at least 1 medical check-up during the previous year. A letter explaining the relevance of depression in diabetes and informing about the study was sent to 4858 patients. A short form of the Patient Health Questionnaire-Depression (PHQ-2), accompanied by a question inquiring into patients’ need to receive professional help with mood-related issues was included, as this modified version was shown to improve the instrument’s specificity [29]. To ensure timely delivery of treatment to potential participants, the questionnaires were sent in six waves. A semi-structured phone interview was used to determine patients’ eligibility based on reporting at least one depressive symptom over the past month, and a need for receiving professional help. The exclusion criteria were major depression or dysthymia, as determined by a phone-administered Structured Clinical Interview for the *Diagnostic and Statistical Manual of Mental Disorders, version IV* (DSM-IV) Axis I disorders (SCID-I) [30], current psychiatric treatment, advanced diabetes complications, and medical contraindications for physical exercise. Patients who did not meet inclusion criteria were informed about other available treatment options, while eligible patients were given basic information about planned treatments, and invited to the clinic for baseline assessment and giving informed consent. At the introductory meeting, patients were informed about the group assignment, their eligibility was confirmed and informed consent obtained, and a 1-hour baseline assessment of body height and weight, questionnaires and laboratory data was carried out. Patients allocated to physical exercise underwent an electrocardiogram (ECG) and, if indicated, an ergometric test.

The participants were recruited from September 2010 to April 2012, and a 1-year follow-up of the latest groups lasted till June 2013. Assessments of psychological and biochemical variables were repeated at 8-week, and at 6 and 12-monthfollow-ups. A 1-year follow-up assessment included a repeated clinical interview to determine hypothetical conversion of depressive symptoms into clinical depression. A more detailed study procedure was described elsewhere [31, 32].

A randomised, repeated measures experimental design was used. Eligible patients willing to participate in the interventions were randomised to psychoeducation and physical exercise or to the enhanced treatment as usual prior to baseline assessment. A computer-generated algorithm stratified by gender [33] provided two lists of random assignments to one of the three groups – one for each stratus – in order to harmonise the number of women and men participating in each group. Research assistants were responsible for assigning the participants to the study arms and conducting the enrolment. No deviations from computer-generated assignments occurred with the exception of one patient who did not accept the proposed assignment. This patient was not included in the study but it was suggested they chose between other sources of professional help.

The outcome assessors were not blinded for the patients’ group assignment, since the included measures (laboratory tests, standardised psychological questionnaires) were not considered likely to cause bias.

Depressive symptoms were primary study outcome, and diabetes-related distress, health-related quality of life, diabetes self-care and metabolic control, as assessed by glycosylated haemoglobin (HbA_1c_) and lipid profile, were secondary outcomes. The sample size calculation was based on the absolute change in depressive symptoms as measured by the Center for Epidemiological Studies Depression Scale (CES-D) questionnaire. An improvement of 0.5 standard deviations was considered clinically relevant [34]. With alpha = 0.05, samples of *n* = 59 per group were shown to be needed to have 80 % power in demonstrating statistically significant differences in depressive symptoms.

### Interventions

#### Psychoeducation

The intervention comprised 6 interactive small-group meetings, each lasting for 90 minutes. The topics included: 1. recognising depressive symptoms; 2. becoming aware of dysfunctional thinking patterns; 3. alleviating the burden of depression through activities and problem solving; 4. understanding cognitive processes that induced and maintained depression; 5. gaining social support, and 6. developing a personal plan for managing mood problems in the future. Meetings at the Outpatient Clinic were held at weekly intervals. The sessions consisted of a short standardised PowerPoint presentation aimed at acquainting patients with basic principles of cognitive behavioural approach to mood problems. The presentation provided a framework for group discussions and a basis for homework assignments. Each session alternated between presentations and discussions on personal experiences, based on the assumption that alternating giving and receiving information would stimulate patients’ active participation. Whenever possible, patients’ problems related to diabetes were used to explore a triad of feelings, thoughts and behaviour.

The participants were provided with a self-help manual for overcoming depressive difficulties based on the “Coping with Depression” course by PM Lewinsohn [35], including a workbook with practical exercises. For the purpose of this study, the programme was adjusted to address specific emotional problems related to diabetes and adapted for a shorter format of this intervention. The manual was tested for comprehensibility and clarity in a group of diabetic patients with different demographic and disease-related characteristics. Patient manual and the presentation used at the sessions are available in Croatian at the Vuk Vrhovac Clinic.

The groups were run by a psychologist experienced in psychoeducation relying on cognitive-behavioural principles.

### Physical exercise

The intervention included 6 weekly 90-minute small-group sessions aimed at educating participants on the interaction between physical activity, mood and diabetes, practising warm-up, flexibility, strengthening and stretching exercises, and at stimulating patients to increase daily physical activities. The sessions combined a short standardised PowerPoint presentation on the topic and practising exercise techniques considered suitable for the participants. Educational topics included: 1. physical activity (PA) in treating diabetes; 2. effects of exercise on glycaemic control and the cardiovascular system; 3. PA and energy expenditure; 4. effects of PA on mobility, muscles and peripheral nerves; 5. effects of exercise on mood; 6. acquiring strategies to maintain physical activities, and developing a personal plan for regular exercise. Educational topics were presented in the first 10–15 minutes of each session including a possibility to exchange personal experiences. The programme was developed and run by a physiotherapist experienced in working with a diabetic population. It was tested within regular educational work with patients in day hospital care.

Exercise intensity was measured by a heart rate monitor and maintained in a light to medium intensity range. Blood glucose and blood pressure were measured before and after each session.

Meetings were held in weekly intervals in a room at the Outpatient Clinic equipped for physical exercise.

Written materials which were given to the patients as a reminder of the practised exercises and the PowerPoint presentation are available in Croatian language at the Vuk Vrhovac Clinic.

Attending at least four sessions of psychoeducation and physical exercise was considered a criterion for receiving the planned treatment.

### Enhanced treatment as usual

No usual care arm was included in the trial because of concerns about leaving patients with depressive symptoms and a need for professional help in a non-interventional study arm. Instead, 1 re-educational intervention of 90 minutes duration was offered. It addressed: a) patients’ understanding of their current HbA_1c_ and lipid values; b) patients’ goals in self-managing diabetes; c) patients’ concerns caused by diabetes in general, and the current laboratory findings. A method of delivery was small-group patient-centred counselling. In addition, patients were provided with written self-help instructions to cope with mood difficulties. This brief intervention was given in a group format and run by a diabetologist experienced in diabetes self-management education.

A summary of programmes’ objectives and contents are delineated in Table 1, while the Template for Intervention Description and Replication (TIDieR) shows the 3 interventions in more detail (see Additional file 1).

**Table 1:** Contents which were discussed and practised within the programmes

Session	Psychoeducation^a^	Physical exercise^b^	Diabetes re-education
1	**Feelings;** recognising and understanding them within the feelings/thoughts/behaviour triad	**Physical activity (PA) in managing diabetes**	Discussing laboratory findings and patients’ satisfaction with them
	**Homework**	**Exercise**	Exchanging experiences with difficulties in managing diabetes
	Keeping mood diary		Distributing leaflets on self-help in mood difficulties
	Paying attention to upward and downward mood spirals		
2	**Thoughts**; becoming aware of hypothetical thinking errors	**Heart and PA**	
	**Homework**	**Exercise**	
	Keeping mood diary and diary of daily activities		
3	**Improving mood by pleasant activities**	**PA and energy expenditure**	
	**Homework**	**Exercise**	
	Developing a plan of pleasant activities, noticing obstacles to accomplishing the plan		
4	**Improving mood by solving problems**	**The effects of PA on motility, muscles and peripheral nerves**	
	**Homework**	**Exercise**	
	Practising a stepped problem-solving technique		
5	**Automatic negative thoughts and how to manage them**	**The effects of PA on mood**	
	**Homework**	**Exercise**	
	Noticing negative thoughts, trying to replace them with more positive ones		
6	**The role of support, and assertive communication**	**Staying motivated for PA**	
	**Developing a plan for self-help in the future**	**Exercise**	

^**a**^Discussing and practising topics focused on diabetes-related issues whenever possible

^**b**^Blood glucose and blood pressure measurements before and at the end of each session. Exercise intensity measured by a heart rate monitor, maintained in a light to medium intensity range

### Assessments

Patient demographic variables included age, sex, education, family status, employment, and economic status. Diabetes-related variables included years of diabetes duration and diabetes treatment with or without insulin.

Screening for mood difficulties was done using the adapted PHQ-2. The participants were asked to indicate if they experienced depressed mood or loss of interest and pleasure in everyday activities in the past month [36]. An additional question, inquiring into the patients’ need for help with these difficulties was added to improve validity [29].

The presence or absence of clinical depression was determined by phone-administered structured clinical interview for the DSM-IV Axis 1 disorders, as this method was demonstrated to be comparable to face-to-face interview [37]. The SCID-I is a semi-structured diagnostic interview that assesses overall psychosocial functioning and presence/absence of clinically significant symptoms. The interview was administered by a trained researcher within the recruitment process and re-administered after 1 year.

Depressive symptoms were measured by the Center for Epidemiological Studies Depression Scale (CES-D), a 20-item self-report instrument [38]. The respondents are asked to indicate the frequency of depressive symptoms in the past week on a four-point scale (0 – rarely or none of the time; 3 – most or all of the time). The total score of the scale ranges from 0 to 60 points, with higher scores indicating higher depressive symptoms. The reliability of the scale at baseline was Cronbach alpha = 0.83.

Diabetes-specific emotional distress was measured by the Problem Areas in Diabetes scale (PAID) [39], a 20-item self-report questionnaire. Respondents are asked to indicate how bothered they are by feelings related to living with diabetes on a 4-point scale (0 – not a problem, 4 – a serious problem). Results are rescaled on a 0–100 scale, with higher scores indicating greater diabetes-related emotional distress. The reliability of the scale at baseline was Cronbach alpha = 0.93.

Diabetes self-care behaviours were measured by the Summary of Diabetes Self-Care Activities (SDSCA) [40]. Respondents assess the number of days per week during which they adhered to dietary recommendations, physical exercise, blood glucose self-monitoring, and foot care. Results are computed as mean values of these subscales, with higher scores indicating better self-care.

Health-related quality of life was measured by the Version 2 of the 12-Item Short Form Health Survey (SF-12v2) [41]. The instrument consists of eight subscales organised into the mental component score (MCS) and the physical component score (PCS). Raw scores are standardised to a 0–100 scale, with higher scores indicating greater quality of life.

HbA_1c_ was measured by an automated immunoturbidimetric assay with dual reporting traceable to National Glycohaemoglobin Standardisation Programme (NGSP) (%) and International Federation of Clinical Chemistry (IFCC) (mmol/mol) reference systems (Integra 400 Tina-quant, Roche, Mannheim, Germany) and a total imprecision, expressed as coefficient of variation (CV) < 2 %. Serum lipids were measured in the morning serum samples, obtained after an overnight fast, by routine automated enzymatic assays (Olympus 680, Beckman Coulter, Brea, CA, USA) with a total imprecision (CV) of 1.16 %, 1.48 % and 1.18 % for the total cholesterol, high-density lipoprotein (HDL)-cholesterol and triglycerides, respectively.

### Statistical methods

One-way analysis of variance (ANOVA) and chi-squares were used to test for baseline differences across the three treatment arms and to examine differences between continuing participants and dropouts. Missing questionnaires’ scores were replaced by average individual scores (CES-D, PAID), or by average group results, in particular subscales (SDSCA, SF-12v2). Replacement was carried out if a number of missing values was not greater than two.

Data were analysed per-protocol, after exclusion of participants who dropped out, died, did not attend all three follow-up meetings or initiated psychopharmacological treatment; these results are presented throughout the manuscript. Intention-to-treat (ITT) analysis was performed to validate these results; all participants who were randomised, with the exclusion of three persons who died during the trial, were analysed despite deviations from protocol. To perform ITT, missing measurements were imputed using the baseline-observation-carried-forward approach.

Repeated-measures ANOVA was used to test for change in outcome variables across time and between the groups. Cohen’s η^2^ coefficients ranging from 0.01–0.05 were regarded small, 0.06–0.12 moderate and ≥ 0.13 large. The outcomes were controlled for gender, age, education, diabetes duration, diabetes treatment, and baseline values of depressive symptoms, diabetes-related distress, HbA_1c_ and low-density lipoprotein (LDL)-cholesterol. If significant interaction between main effects and covariates occurred, separate ANOVA models for divided subgroups were employed. Post-hoc ANOVA’s comparing psychological-related and diabetes-related outcomes in patients with high versus low baseline values of depressive symptoms, diabetes distress, HbA_1c_ and cholesterol were used to test relative benefits of the interventions.

A *p* value of < 0.05 was considered significant in all analyses. Statistical analyses were performed by using the SPSS 17 (SPSS Inc., Chicago, IL, USA)

### Ethical aspects

The study was approved by the Vuk Vrhovac University Clinic for Diabetes Ethics Committee on 30 June 2010.

## Results

Among the 4858 patients identified from the registry of diabetic patients on the basis of the inclusion criteria, 365 were considered eligible and 265 agreed to participate in the interventions (72 %); 209 of them completed baseline laboratory and psychological assessments (79 %), with 74 randomised to psychoeducational intervention, 66 to physical exercise and 69 to diabetes re-education. Fifty-six patients withdrew their previous agreement to participate because of missing baseline assessments or reporting competitive life demands as an obstacle.

**Fig. 1:**
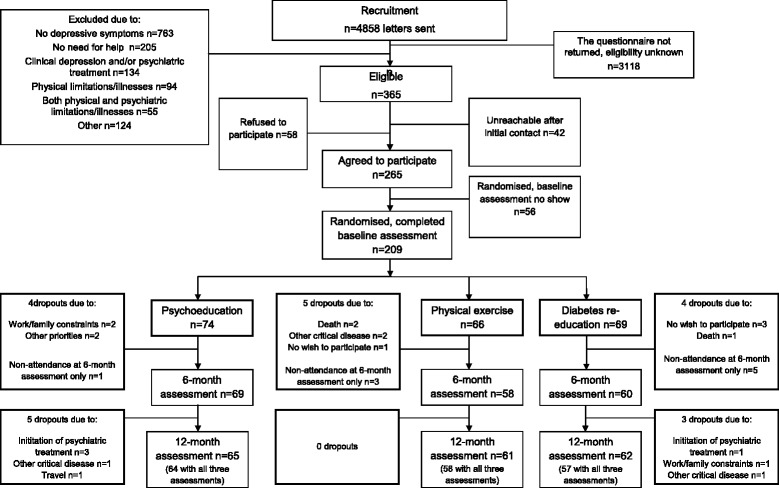
Recruitment and retention flow diagram

Attrition rate was 3 % during the interventions (6 of 209 patients), 3 % from baseline to 6-months (7 of 203 patients), and 2 % from 6-month to 12-month follow-up period (4 of 196 patients). Of the seven dropouts who completed the intervention but missed both follow-up assessments, two missed the follow-up appointments due to health problems, four were unwilling to come and one patient died. Four patients were excluded from per-protocol analyses due to the initiation of pharmacological therapy or discovery of psychiatric co-morbidities that were not reported during the recruitment period. No differences between the participants and dropouts across the three study groups were observed. The patients’ flow is presented in Fig. 1.

Table 2 presents demographic, diabetes-related and psychological characteristics of patients randomised to one of the 3 groups. No between-group differences were found, with the exception of self-reported healthy eating as measured by the SDSCA, which was better in the psychoeducational group (*p* = 0.012).

**Table 2:** Baseline characteristics of participants by the intervention groups (*n* = 209)

	Psychoeducation	Physical exercise	Diabetes re-education	Significance
n	74	66	69	
Age	57.7 (6.2)	58.5 (4.8)	58.2 (5.6)	0.648
Women	40	37	36	0.867
Education (years)	12.6 (2.4)	12.4 2.3)	12.2 (2.5)	0.623
Professional status	30	27	30	0.502
(% employed)				
Economic status	13	12	13	0.317
(% poor)				
Family status	76	72	76	0.488
(% married/cohabitating)				
Diabetes duration (years)	11.4 (9.1)	12.9 (2.8)	10.5 (6.9)	0.283
Insulin use	32	29	32	0.695
Body mass index kg/m^2^	30.64 (4.54)	29.44 (4.67)	29.96 (4.39)	0.297
HbA_1C_ (%)	7.4 (1.2)	7.2 (1.1)	7.2 (1.1)	0.384
HbA_1C_ (mmol/mol)	58 (13)	56 (13)	55 (11)	0.442
Cholesterol (mmol/l)	5.3 (1.2)	5.3 (1.1)	5.3 (1.1)	0.903
Depressive symptoms	19.7 (9.1)	20.5 (8.6)	19.7 (8.7)	0.813
(CES-D score)				
Diabetes distress	37.9 (19.7)	42.6 (20.5)	39.1 (19.6)	0.358
(PAID score)				
Health-related QoL (SF-12v2)				
Mental component score	41.9 (7.4)	41.7 (8.3)	41.2 (7.2)	0.872
Physical component score	42.3 (8.7)	43.1 (8.8)	42.7 (9.1)	0.871
Healthy eating				
Diabetic diet	4.1 (1.5)	3.8 (1.5)	3.4 (1.6)	0.012
Exercise	3.9 (1.8)	3.5 (1.9)	3.4 (1.9)	0.189
Blood glucose self-monitoring	2.9 (1.7)	2.5 (1.9)	2.6 (1.6)	0.401
Foot care	3.9 (2.5)	3.5 (2.9)	3.6 (2.6)	0.666
(SDSCA scores)	2.5 (2.4)	2.4 (2.2)	2.9 (2.5)	0.341

Data are means (SD) or percentages

One-way ANOVA or *χ* ^2^ as appropriate

*CES-D* Centre for Epidemiological Studies-Depression scale, *HbA*_*1c*_ glycosylated haemoglobin, *PAID* Problem Areas in Diabetes scale, *QoL* quality of life, *SDSCA* SDSCA, Summary of Diabetes Self-Care Activities, *SF-12v2* Version 2 of the 12-Item Short Form Health Survey

The effects of interventions on psychological, behavioural and metabolic outcomes based on repeated measures ANOVA are given in Table 3. ITT analyses showed a comparable pattern of results of all analyses in terms of statistical significance and effect size, and are not presented in the text.

**Table 3:** One-year changes in psychological, behavioural and metabolic outcome variables

	Group scores	Baseline M (SD)	12-month follow-up M (SD)	Effects	F	Overall *p* ^a^	*p* baseline to 12 months^b^	η^2^
Psychological outcomes	Depressive symptoms (CES-D)							
	A (*n* = 64)	19.7 (9.1)	16.7 (7.9)	Time	12.51	<0.001	0.003	0.07
	B (*n* = 58)	19.8 (8.2)	18.1 (9.8)	Time x group	0.609	0.656		0.007
	C (*n* = 57)	19.0 (8.6)	17.4 (9.0)					
	Diabetes-related problems							
	(PAID)							
	A (*n* = 64)	37.6 (20.2)	32.5 (22.1)	Time	16.87	<0.001	<0.001	0.09
	B (*n* = 57)	41.8 (20.9)	36.4 (22.1)	Time x group	0.389	0.796		0.004
	C (*n* = 57)	38.0 (18.6)	33.2 (20.3)					
	Quality of life (SF-12v2)							
	Mental component score (MCS)							
	A (*n* = 64)	41.4 (6.7)	45.1 (6.7)	Time	18.13	<0.001	<0.001	0.09
	B (*n* = 58)	41.9 (8.0)	44.7 (8.1)	Time x group	1.25	0.288		0.014
	C (*n* = 56)	41.6 (7.3)	43.7 (8.5)					
Behavioural outcomes	Diabetes self-care (SDSCA)							
	Healthy eating							
	A (*n* = 62)	4.2 (1.4)	4.2 (1.3)	Time	4.78	0.009	0.01	0.03
	B (*n* = 56)	3.7 (1.4)	4.1 (1.5)	Time x group	1.66	0.158		0.019
	C (*n* = 56)	3.4 (1.6)	4.0 (1.3)					
	Diabetic diet							
	A (*n* = 60)	3.9 (1.7)	3.9 (1.7)	Time	4.58	0.01	0.02	0.03
	B (*n* = 57)	3.5 (1.9)	4.3 (1.7)	Time x group	1.65	0.165		0.019
	C (*n* = 56)	3.4 (2.1)	3.7 (1.9)					
	Physical exercise							
	A (*n* = 64)	2.8 (1.7)	3.2 (1.9)	Time	5.14	0.006	0.008	0.03
	B (*n* = 57)	2.5 (2.0)	3.1 (1.5)	Time x group	0.375	0.826		0.004
	C (*n* = 57)	2.4 (1.6)	2.6 (1.7)					
	Blood glucose self-monitoring							
	A (*n* = 63)	3.7 (2.5)	4.9 (2.1)	Time	16.29	<0.001	<0.001	0.09
	B (n = 56)	3.7 (2.9)	4.3 (2.2)	Time x group	0.589	0.664		0.007
	C (n = 57)	3.6 (2.7)	4.5 (2.3)					
	Foot self-care							
	A (*n* = 64)	2.5 (2.2)	3.2 (2.2)	Time	15.92	<.001	<0.001	0.08
	B (*n* = 57)	2.3 (2.2)	3.1 (2.2)	Time x group	0.373	0.809		0.004
	C (*n* = 57)	2.8 (2.4)	3.3 (2.3)					
Metabolic outcomes	HbA_1C_ (%)/(mmol/mol)							
	A (*n* = 64)	7.4/58 (1.3/14)	7.2/56 (0.9/10)	Time	2.70	0.079	0.683	0.015
	B (*n* = 58)	7.2/55 (1.0/11)	7.2/56 (1.0/11)	Time x group	0.922	0.438		0.010
	C (*n* = 57)	7.1/55 (1.0/11)	7.0/54 (1.0/11)					
	Total cholesterol (mmol/l)							
	A (*n* = 64)	5.3 (1.1)	4.9 (1.0)	Time	11.64	<0.001	<0.001	0.06
	B (*n* = 58)	5.2 (1.4)	5.0 (1.3)	Time x group	0.684	0.604		0.008
	C (*n* = 57)	5.3 (1.1)	4.9 (1.0)					
	LDL-cholesterol (mmol/l)							
	A (*n* = 64)	3.1 (1.0)	2.8 (0.8)	Time	6.50	0.002	0.003	0.04
	B (*n* = 58)	2.9 (1.2)	2.8 (1.1)	Time x group	1.55	0.188		0.017
	C (*n* = 57)	3.0 (1.0)	2.7 (0.8)					
	HDL-cholesterol (mmol/l)							
	A (*n* = 64)	1.4 (0.3)	1.3 (0.3)	Time	9.56	<0.001	<0.001	0.05
	B (*n* = 58)	1.4 (0.4)	1.3 (0.3)	Time x group	0.196	0.929		0.002
	C (*n* = 57)	1.4 (0.3)	1.4 (0.3)					
	Triglycerides (mmol/l)							
	A (*n* = 64)	2.1 (1.6)	1.8 (0.8)	Time	6.58	0.003	0.010	0.04
	B (*n* = 58)	2.1 (1.2)	1.9 (1.0)	Time x group	0.194	0.911		0.002
	C (*n* = 57)	2.1 (1.7)	1.8 (0.8)					

^a^two-sided significance of differences across time and between the groups (repeated measures ANOVA)

^b^two-sided significance of differences between baseline assessment- and 12-month assessment

*CES-D* Centre for Epidemiological Studies-Depression scale, *HbA*_*1c*_ glycosylated haemoglobin, *HDL* high-density lipoprotein, *LDL* low-density lipoprotein *PAID* Problem Areas in Diabetes scale *SDSCA* Summary of Diabetes Self-Care Activities

### Changes in depressive symptoms, diabetes-related distress and health-related quality of life

No significant between-subject effects were obtained with respect to depressive symptoms, diabetes distress and health-related quality of life (*p* = 0.656, *p* = 0.796, and *p* = 0.288, respectively), indicating comparable efficacy of psychoeducation, physical exercise and enhanced treatment as usual on these outcomes.

Significant reductions in depressive symptoms occurred across all 3 study groups from baseline to 12 months (F = 12.51, *p* < 0.001, η^2^ = 0.7). The size of the time effect indicated a moderate effect of the three treatments on the primary study outcome.

Statistically significant time effects of interventions on diabetes-related distress (F = 16.87, *p* < 0.001, η^2^ = 0.09) occurred in all 3 groups from baseline to 12 months.

Mental component of health-related quality of life improved significantly in all treated individuals (F = 18.13, *p* < 0.001, η^2^ = 0.09) during the 1-year follow-up period, while physical quality of life aspect remained unchanged (*p* = 0.711).

The results remained significant after controlling for age, education, diabetes duration and treatment.

### Changes in behavioural outcomes (diabetes self-care activities)

No significant between-subject effects were obtained with respect to self-care behaviours as determined by self-reported healthy eating, maintaining a diabetic diet, exercising, blood glucose self-monitoring and foot self-care (SDSCA; all *p* values > 0.05).

A small effect of interventions on healthy eating and diabetic diet (F = 4.78, *p* = 0.01, η^2^ = 0.03; and F = 4.58, *p* = 0.02, η^2^ = 0.03, respectively) occurred in all 3 groups from baseline to 12 months, as did self-reported exercising (F = 5.14, *p* = 0.008, η^2^ = 0.03). Moderate main time effects were registered with respect to blood glucose self-monitoring and foot self-care (F = 16.29, *p* < 0.001, η^2^ = 0.09; and F = 15.92, *p* < 0.001, η^2^ = 0.08, respectively).

### Changes in metabolic outcomes

No significant between-subject effects occurred with respect to glycaemic control and lipid profile (all *p* values > 0.05).

In all treated individuals, changes in HbA_1c_ across time were of borderline significance (F = 2.70, *p* = 0.078), while changes in total cholesterol, LDL-cholesterol and triglycerides reached significant and clinically meaningful improvement during the 1-year follow-up (F = 11.64, *p* < 0.001; F = 6.50, *p* = 0.002; and F = 6.58, *p* = 0.003, respectively).

The results remain significant after controlling for changes in lipid-lowering therapy during the follow-up period.

### Changes in psychological and metabolic outcomes in relation to baseline symptoms and control level

Time changes of psychological and metabolic variables in the 2 subgroups of patients – with baseline CES-D and PAID scores above/below cut-offs of 16 and 40, and with baseline HbA_1c_ higher/lower than 7 % (53 mmol/mol) and LDL-cholesterol higher/lower than 2.6 mmol/l – are presented in Table 4.

**Table 4:** One-year changes in psychological and metabolic outcomes in the subgroups of patients with low versus high baseline values

	Baseline values	Number	M (SD) Baseline to 12 months		Mean differences (95 % CI) Baseline to 12 months	Effects	F	*p* ^a^	*p* baseline to 12 months^b^	η^2^
Depressive symptoms										
(CES-D)	<16	62	10.8 (3.4)	12.0 (6.8)	1.22 (−0.82, 3.25)	Time	1.24	0.293	0.435	0.02
						Time x group	0.99	0.412		
	≥16	117	24.1 (6.8)	20.2 (8.8)	−3.91 (−5.89, −1.93)	Time	21.27	<0.001	<0.001	0.16
						Time x group	1.58	0.179		
Diabetes distress (PAID)										
	<40	94	23.6 (10.2)	24.0 (17.2)	0.47 (−3.28, 4.22)	Time	0.98	0.371	1.00	0.01
						Time x group	0.62	0.637		
	≥40	84	56.4 (12.3)	45.1 (20.3)	−11.51 (−16.05, −6.97)	Time	29.57	<0.001	<0.001	0.27
						Time x group	1.10	0.356		
HbA_1c_, (mmol/mol)	<53	79	46 (5)	50 (9)	3.95 (1.72, 6.19)	Time	15.05	<0.001	<0.001	0.16
						Time x group	0.58	0.610		
	≥53	100	64 (10)	60 (10)	−4.27 (−7.10, −1.46)	Time	13.38	<0.0001	<0.001	0.12
						Time x group	1.25	0.295		
HbA_1c_ (%)	<7.0	80	6.3 (0.5)	6.6 (0.8)	0.34 (0.14, 0.54)	Time	13.89	<0.001	<0.001	0.15
						Time x group	0.52	0.662		
	≥7.0	99	8.0 (0.9)	7.6 (0.9)	−0.44 (−0.70, −0.18)	Time	14.51	<.001	<.001	0.13
						Time x group	1.59	0.189		
LDL-cholesterol (mmol/l)										
	<2.3	44	1.8 (0.4)	2.1 (0.7)	0.25 (0.001, 0.50)	Time	5.52	0.009	0.05	0.12
						Time x group	1.31	0.279		
	≥2.3	135	3.4 (0.9)	3.0 (0.9)	−0.38 (−0.56, −0.19)	Time	12.22	<0.001	<0.001	0.09
						Time x group	1.24	0.295		

^a^two-sided significance of differences across time and between the groups (repeated measures ANOVA)

^b^two-sided significance of differences between baseline- and 12-month assessments

*CES-D* Centre for Epidemiological Studies-Depression scale, *HbA*_*1c*_ glycosylated haemoglobin, *LDL* low-density lipoprotein, *PAID* Problem Areas in Diabetes scale

Emotional symptoms in individuals with milder depressive symptoms (CES-D < 16) and no serious problems related to diabetes (PAID < 40) did not improve but remained within subclinical scores’ range. Patients with baseline CES-D and PAID scores above the cut-points, however, demonstrated continuous and clinically relevant improvement over a one-year period (η^2^ = 0.16 and η^2^ = 0.27, respectively).

The same pattern of change was observed with respect to metabolic indicators. Patients with baseline HbA_1c_ values lower than 7 % (<53 mmol/mol), and LDL-cholesterol lower than 2.6 mmol/l remained within reference values over a 1-year follow-up period, although variability of these indicators was shown to be statistically significant. Patients with unsatisfactory metabolic control at baseline reached significant and clinically relevant improvement in both glycaemic and lipid control (η^2^ = 0.13 and η^2^ = 0.09, respectively).

## Discussion

The main finding of this 1-year study was that subclinically depressed patients treated by psychoeducation, physical exercise and enhanced treatment as usual comparably improved depressive symptoms, diabetes distress, self-management of diabetes, health-related quality of life and metabolic control. The absence of a significant treatment effect suggested that patterns of change might be similar in all treatment groups. Psychoeducation and physical exercise as more complex treatments were not superior to enhanced treatment as usual – a short diabetes re-education focused on patients’ diabetes-related concerns, but were equally effective.

This might be associated with the nature of depressive symptoms in the treated individuals on the one hand, and with inter-relations of depressive symptoms and diabetes-related distress on the other hand. Mild depression, common in all treated individuals, could imply that they were only partially aware of having a problem, which is relevant and can be approached actively. Once clinical attention to their difficulties was established, patients’ self-awareness might be increased and their readiness to put efforts into a more active approach empowered, regardless whether a structured anti-depressive programme or an unspecific support was offered.

In the study participants, elevated depressive symptoms were strongly associated with diabetes distress (r = 0.353, *p* < 0.01) and 53 % also reported serious problems caused by diabetes. As psychoeducational and physical exercise treatments tended to focus on diabetes-related problems as did the enhanced treatment as usual, addressing diabetes distress can be supposed to be a pathway likely to improve outcomes in all three groups.

The obtained results can be put into a context of the study by Fisher et al. [20] on treatment of diabetes distress, which found that significant and clinically meaningful reductions in emotional symptoms and improvement in self-management behaviours occurred in three different treatment conditions, one of which being minimal. As interpreted, patients with mild symptoms are highly responsive to clinical staff attention, concern and support, both with and without any structured programme to alleviate their emotional problems. Similar mechanisms of change could be hypothesised in our study. Patients in the enhanced treatment as usual, which consisted of one re-educational session, did not benefit only from this intervention but also from the entire trial procedure, which comprehended assessing individual problems, structuring diabetes care more strictly and maintaining periodical phone contacts. Although the exact sources of this non-specific support and their associations with patients’ feelings and behaviours can only be hypothesized, knowing that even minimal intensification of diabetes treatment is helpful in individuals who suffer from subclinical depression is relevant to everyday clinical practice. This is especially true considering the high prevalence of elevated depressive symptoms in patients with diabetes, as well as the necessity for interventions capable of achieving high reach and not requiring complex prerequisites for implementation.

A comparable improvement in depressive symptoms in all treatment groups might be hypothesised to be attributable to spontaneous remission of depression. However, reports on the persistence of elevated depressive symptoms in diabetic population [42, 43], as well as the findings on increased risk of developing major depression once diabetic patients are faced with elevated depressive symptoms [13], do not suggest that spontaneous recovery would occur in such a systematic way.

Another relevant finding of this study is that even minimally intensified treatment as usual was associated with changes on multiple levels, including psychological, behavioural and biochemical ones, and continued over time. The effects remained significant after controlling for age, education, diabetes duration and insulin treatment, suggesting that the findings may be generalised to most type 2 diabetes patients suffering from subsyndromal depression.

The improvement in emotional symptoms and metabolic control is even more apparent when viewing the subgroups of patients with higher depressive symptoms, more serious emotional problems related to diabetes and suboptimal metabolic control at baseline. Compared with patients with milder baseline symptoms and satisfactory metabolic control, these patients achieved greater reduction in depressive symptoms and diabetes distress, and reached significant and clinically meaningful improvement in HbA_1c_ and LDL-cholesterol. In contrast, patients who were below standard cut-offs at baseline, remained so during the 1 year follow-up. The latter corresponds with well-known findings that individuals who entered a programme with better values on a variety of indicators improve less on these indicators [44]. However, the importance of including subclinically depressed patients with even mild symptoms into behavioural interventions, especially if they express a need to receive professional help in mood-related issues, may be supported by avoiding worsening subjective symptoms proven to be likely in these individuals [13].

Reviews on treating subclinical depression in the general population [45] report positive, but modest size effects, which are not maintained during a follow-up period. Also, insufficient evidence has been found to support the use of psychological interventions in subclinical depression to prevent the emergence of major depression [46]. Our results are promising with respect to all the mentioned problem areas. The obtained changes of subjective and objective symptoms, as well as their persistence over time, can be considered clinically meaningful. In addition, none of the included patients developed major depressive disorder over a 1-year period. Although four patients were excluded from the analysis because of starting antidepressant treatment during the follow-up period, the repeated SCID-I interview did not confirm major depressive disorder but rather the patients’ preference for pharmacotherapy.

This study has several strengths. It was focused on highly prevalent population of type 2 diabetes patients with depressive symptoms but no clinical depression, relied on a randomised comparative design, investigated samples large enough to demonstrate statistically significant changes, and used structured interventions with reasonable requirements in terms of staff, duration and costs. Its limitation is the lack of possibility to investigate the course and outcomes of subclinical depression without any treatment, as no usual care was included in the trial because of concerns about leaving patients without professional help. Also, the study was carried out in only one centre – a tertiary diabetes clinic – which does not allow concluding about its feasibility in other clinical settings. Allocating patients to the treatment groups was not concealed to researchers and the outcome assessors were not blinded. Both could be considered as the study weaknesses although a computer-generated randomisation, and highly standardised outcome measures substantially reduce hypothetical source of bias.

## Conclusions

Depressive symptoms that are not severe enough to warrant a diagnosis of clinical depression are highly prevalent in diabetic population and are associated with both poor well-being and poor diabetes self-management. Our study showed that psychoeducation, physical exercise and enhanced treatment as usual had comparable effects on long-term improvement of psychological, behavioural and metabolic outcome suggesting that even minimal intervention addressing patients’ diabetes-related problems and concerns had favourable clinical implications. Further investigation is warranted to clarify possible mechanisms of improvement.

## Additional file

Additional file 1:
**The Template for Intervention Description and Replication (TIDieR) checklist.**

## Abbreviations

ANOVA: analysis of variance
CES-D: Centre for Epidemiological Studies-Depression scale
CV: coefficient of variation
DSM-IV: *Diagnostic and Statistical Manual of Mental Disorders, version IV*
ECG: electrocardiogram
HbA_1c_: glycosylated haemoglobin
HDL: high-density lipoprotein
IFCC: International Federation of Clinical Chemistry
LDL: low-density lipoprotein
MCS: mental component score
NGSP: National Glycohaemoglobin Standardisation Programme
PA: physical activity
PAID: Problem Areas in Diabetes scale
PCS: physical component score
PHQ-2: Patient Health Questionnaire-Depression
QoL: quality of life
SCID-I: Structured Clinical Interview for the DSM-IV Axis I disorders
SDSCA: Summary of Diabetes Self-Care Activities
SF-12v2: Version 2 of the 12-Item Short Form Health Survey
TIDieR: Template for Intervention Description and Replication
