# Supplementary material for: Does treatment of subsyndromal depression improve depression-related and diabetes-related outcomes? A randomised controlled comparison of psychoeducation, physical exercise and enhanced treatment as usual
Source: Trials. 2015 Jul 15;16:305. doi: 10.1186/s13063-015-0833-8 (PMC4501064; doi:10.1186/s13063-015-0833-8)
Supplement: Additional file 1: — The Template for Intervention Description and Replication (TIDieR) checklist. [file 13063_2015_833_MOESM1_ESM.docx]

**The Template for Intervention Description and Replication (TIDieR) checklist:**

**Psychoeducation and physical exercise compared to enhanced treatment as usual in type 2 diabetic patients with subsyndromal depression**

| Brief name |  |  |  |
| --- | --- | --- | --- |
| 1 | **Psychoeducation** | **Physical exercise** | **Enhanced treatment as usual** |
| Why |  |  |  |
| 2 | **Treatment based on cognitive behavioural principles was proven to be effective in depressed patients with diabetes [1]. The psychoeducational course using cognitive behavioural techniques was supposed to represent a feasible intervention in routine clinical practice. Due to its relative simplicity, it was expected to be suitable for reaching a substantial number of patients with subsyndromal depression which is in accordance with the RE-AIM recommendations for evaluating impacts of self-care interventions in diabetes [2].** | **At the time of starting this RCT (September 2010), there were no interventional studies in type 2 diabetic patients examining the impact of a physical activity intervention on mood. Nonetheless, the positive association between depressed mood and inactivity found in cross-sectional studies suggested that physical activity interventions may have the potential to improve mood in depressed diabetic patients.** | **No usual care arm was included in the trial because of concerns about leaving patients with depressive symptoms and a need for professional help in a non-interventional study arm. Instead, one re-educational intervention lasting for 90 minutes was offered.** |
| What |  |  |  |
| 3 | **Patients included into psychoeducational course received six group sessions aimed at acquiring CBT techniques to self-manage mood difficulties. A learning process was facilitated by PowerPoint presentations explaining the importance of recognising depressive symptoms, dysfunctional thinking patterns and automatic negative thoughts, and alleviating symptoms through activities, problem solving techniques and assertive communication. The participants were provided with a self-help manual for overcoming depressive difficulties based on the “Coping with depression” course by P.M. Lewinsohn [3,4], including a workbook with practical exercises. For the purpose of this study, the programme was adjusted to address specific emotional problems related to diabetes and adapted for a shorter format of this intervention. Patient manual and the presentation used at the sessions are available in Croatian language at the Vuk Vrhovac Clinic.** | **Patients included into physical exercise received six weekly small group sessions, aimed at educating participants on the interaction between physical activity, mood and diabetes, practicing warm-up, flexibility, strengthening and stretching exercises, and at stimulating patients to increase daily physical activities. The sessions combined a short standardised PowerPoint presentation on the topic and practicing exercise techniques considered suitable for the participants. Written materials which were given to the patients as a reminder of the practiced exercises and the PowerPoint presentation are available in Croatian language at the Vuk Vrhovac Clinic.** | **Patients included into enhanced treatment as usual received one** **re-educational intervention. It addressed patients’ understanding of their current HbA1C and lipid values, patients’ goals in self-managing diabetes and patients’ concerns caused by diabetes in general, and the current laboratory findings. In addition, patients were provided with written self-help instructions to cope with mood difficulties. The latter materials are available in Croatian language at the Vuk Vrhovac Clinic.** |
| 4 | **The first session started with welcoming patients and explaining the content of the psychoeducational course (What shall we learn during this course?) followed by the first PowerPoint presentation entitled Feelings. In order to stimulate patients’ active participation, presenting materials alternated with discussing personal experiences. The following sessions were entitled: Thinking errors; Activities; Problem solving; Automatic negative thoughts, and Support (from others and themselves - the role of assertive communication). Each session ended with agreeing on a homework assignment which served as a basis for group discussion at the beginning of the next session.** | **The first session started with welcoming patients and explaining the content of the course. Six PowerPoint presentations were prepared:** **Physical exercise (PA) and diabetes; PA and glycaemic control; PA and energy expenditure; PA, mobility, muscles and peripheral nerves; PA and mood; Planning and maintaining PA. Presentations lasted for 10-15 minutes followed by warm-up, flexibility, strengthening and stretching exercises.** **Blood glucose and blood pressure were measured before and after each session. Exercise intensity was measured by a heart rate monitor and maintained in a light to medium intensity range.** | **The session started with welcoming patients and stressing the importance of an active approach to diabetes-related difficulties. A patient-centred counselling in a group was used to address: patients’ understanding of their current HbA1c and lipid values; patients’ goals in self-managing diabetes; patients’ concerns caused by diabetes in general, and the current laboratory findings. Patients were encouraged to develop a personally acceptable plan to self-manage diabetes effectively.** |
| Who provided |  |  |  |
| 5 | **The programme was developed and run by a psychologist experienced in psychoeducation relying on cognitive-behavioural principles.** | **The programme was developed and run by a physiotherapist experienced in working with diabetic population.** | **This brief intervention was run by a diabetologist experienced in diabetes self-management education.** |
| How |  |  |  |
| 6 | **The sessions were given in a group format, each group consisting of 5-8 patients, harmonized with respect to gender.** | **The sessions were given in a group format, each group consisting of 5-8 patients, harmonized with respect to gender.** | **The session was given in a group format, each group consisting of 5-8 patients, harmonized with respect to gender.** |
| Where |  |  |  |
| 7 | **Sessions were held outside regular working hours at the Outpatient clinic. The room, generally used for patient education, provided appropriate prerequisites for running a group - comfort, possibilities for exchanging verbal and non-verbal messages, privacy.** | **Sessions were held outside regular working hours at the Outpatient clinic appropriately equipped for physical exercise.** | **The session was held outside regular working hours at the Outpatient clinic, in the room intended for patient education.** |
| When and how much |  |  |  |
| 8 | **There were six sessions, each lasting for 90 minutes, which were delivered in the afternoon, outside regular working hours.** | **There were six sessions, each lasting for 90 minutes, which were delivered in the afternoon, outside regular working hours.** | **There was one session lasting for 90 minutes, which was delivered in the afternoon, outside regular working hours.** |
| Tailoring |  |  |  |
| 9 | **The intervention was planned to be the same for all participants.** | **The intervention was planned to be the same for all participants.** | **The intervention was planned to be the same for all participants.** |
| Modifications |  |  |  |
| 10 | **The intervention was not modified during the course.** | **The intervention was not modified during the course.** | **The intervention was not modified during the course.** |
| How well |  |  |  |
| 11 | **The intervention was delivered by a health professional (psychologist) with expertise in the applied approach. The intervention fidelity was not assessed.** | **The intervention was delivered by a health professional (physiotherapist) with expertise in diabetes, physical activity and running educational groups. The intervention fidelity was not assessed.** | **The intervention was delivered by a health professional (physician) with expertise in patient education. The intervention fidelity was not assessed.** |
| 12 |  |  |  |
|  | **NA** | **NA** | **NA** |

**Citation:**

1. Penchofer SM, Ferrans C, Mumby M, Byrn M, Emanuele MA, Harrison PR, Durazo-Arvizu RA, Lustman P. A Psychoeducational Intervention (SWEEP) for Depressed Women with Diabetes. Ann Behav Med 2012; 44: 192-206.
2. Glasgow RE, Vogt TM, Boles SM. Evaluating the public health impact of health promotion interventions: the RE-AIM framework. Am J Pub Health 1999; 89: 1322-1327.
3. Lewinsohn PM. The Coping with depression course: a psychoeducational intervention for unipolar depression. Eugene, OR: Castalia Pub. C0. 1084
4. Cuijpers P, Munoz RF, Clarke GN, Lewinsohn PM. Psychoeducational treatment and prevention of depression: the "Coping with Depression" course thirty years later. Clin Psychol Rev 2009; 29: 449-458
